# Intestinal Obstruction Caused by a Fruit Seed: A Rare Case Without Gastrointestinal Disease

**DOI:** 10.7759/cureus.3767

**Published:** 2018-12-21

**Authors:** Takuma Iwai, Hiroshi Makino, Tadashi Yokoyama, Hiroshi Maruyama, Atsushi Hirakata, Toshimitsu Miyasaka, Hiroshi Yoshida

**Affiliations:** 1 Surgery, Nippon Medical School, Tokyo, JPN; 2 Oncology, Nippon Medical School, Tokyo, JPN

**Keywords:** sbo, intestinal obstruction, foreign body, seed

## Abstract

We report here a rare case of intestinal obstruction caused by a peach seed. A 15-year-old boy was admitted to our hospital because of abdominal pain and vomiting. The patient had no history of previous gastrointestinal surgery and his medical comorbidity was autism. A computed tomography (CT) scan showed an obstruction of the ileum by a foreign body. Surgical treatment was successfully performed, and we found a peach seed in the ileum. He was discharged eight days after the operation without postoperative complications. Intestinal obstruction caused by plant seeds without gastrointestinal disease is rare.

## Introduction

Intestinal obstruction is a common disease that is encountered in surgical practice. Intestinal obstructions are often induced by adhesion, hernia, neoplasm, and inflammation, but intestinal obstruction due to feeding is rare [1]. Furthermore, plant seeds that pass through the pylorus do not induce intestinal obstruction because swallowed seeds are often small and ovoid shaped [2-3]. Therefore, in most cases of intestinal obstruction caused by plant seeds, the patients have gastrointestinal disease such as a malignant tumor and postoperative adhesion.

We describe a patient without gastrointestinal disease in whom a peach seed induced small bowel obstruction. The seed was successfully removed with surgical treatment.

## Case presentation

A 15-year-old boy was admitted to Nippon Medical School Tama Nagayama Hospital because of appetite loss, vomiting, and abdominal pain persisting for approximately seven days. The patient had no history of previous gastrointestinal surgery and his medical comorbidity was autism. His vital parameters were normal and his general physical examination results were unremarkable. An abdominal examination showed distention and mild generalized tenderness without signs of peritonitis. Laboratory studies showed a mild elevation of the white blood cell count (8100/µl), a serum C-reactive protein level of 1.61 mg dl, and a serum total bilirubin level of 1.9 mg/dl (Table 1).

**Table 1:** Laboratory data on admission White blood cell (WBC), red blood cell (RBC), Hemoglobin (Hb), platelet (Plt), prothrombin time (PT), activated partial thromboplastin time (APTT), aspartic aminotransferase (AST), alanine transaminase (ALT), lactate dehydrogenase (LDH), alkaline phosphatase(ALP), γ-glutamyltransferase (γGTP), amylase (AMY), total-bilirubin (T-bil), total protein (TP), albumin (Alb), blood urea nitrogen (BUN), creatinine (Cre), C-reactive protein (CRP)

	Laboratory data on admission					
WBC	8100	/ul		AST	22	IU/l
RBC	556×10^4^	/ul		ALT	14	IU/l
Hb	16.7	g/dl		LDH	198	IU/l
Ht	43.6	%		ALP	167	IU/l
Plt	26.3	/ul		γGTP	13	IU/l
				AMY	53	IU/l
				T-bil	1.9	mg/dl
				TP	7.5	g/dl
PT	59.5	%		Alb	4.8	g/dl
APTT	28.4	sec		BUN	17.2	mg/dl
				Cre	0.69	mg/dl
				Na	133	mEq/l
				K	4.1	mEq/l
				Cl	92	mEq/l
				CRP	1.61	mg/ml

**Figure 1:**
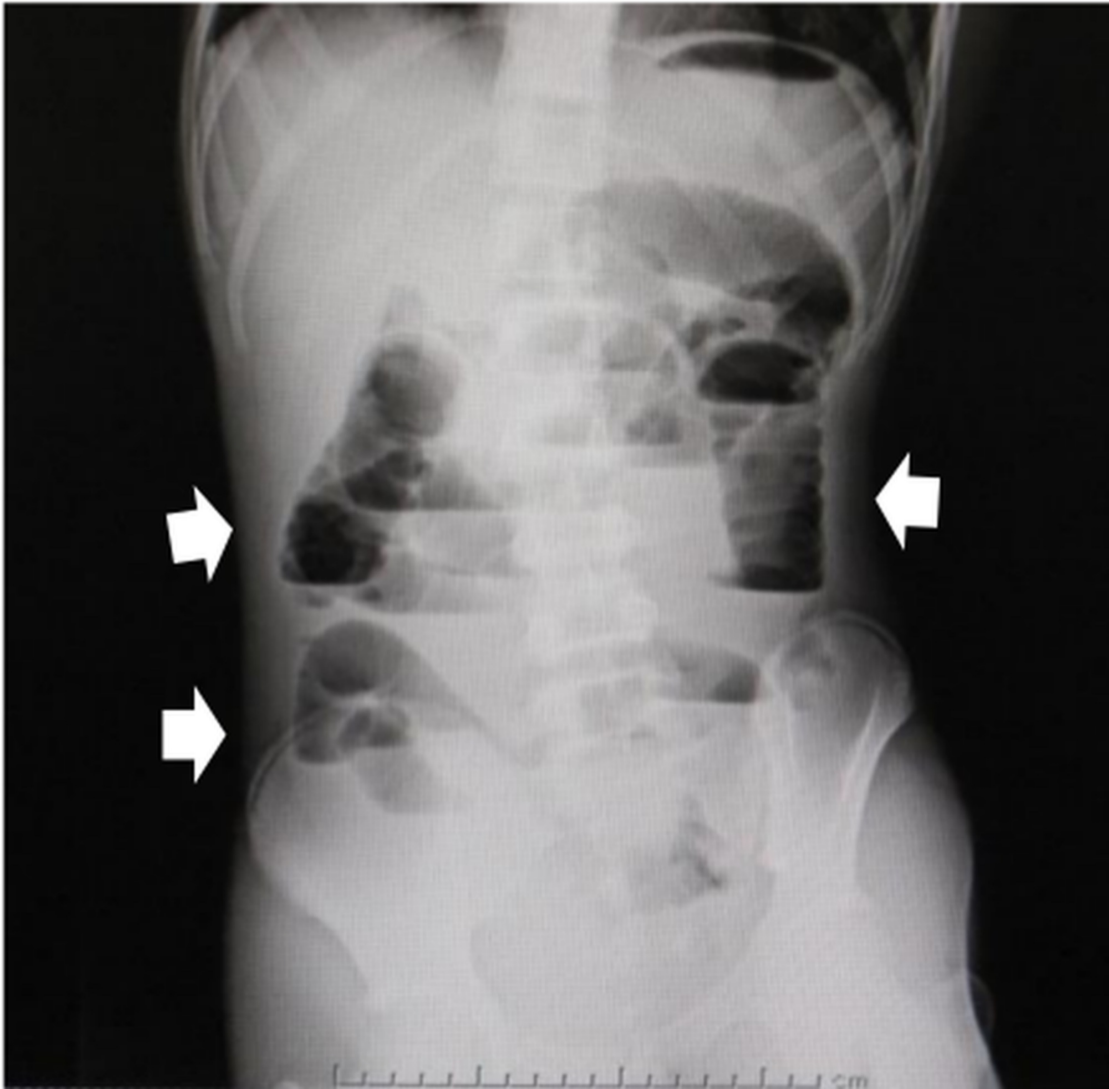
Abdominal X-ray Plain radiography shows dilatation and a stair-step pattern of the small intestine.

An abdominal X-ray demonstrated dilatation and a stair-step pattern in the small intestine (Figure 1).

**Figure 2:**
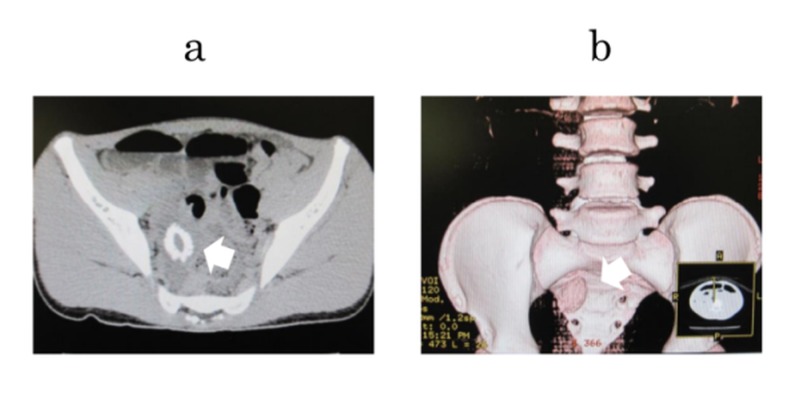
Abdominal CT A foreign body was detected in the small intestine (arrow). The shape of the object was oval. a: Coronal plain abdominal CT. b: Three-dimensional construction from the CT images. CT: computed tomography

Computed tomography (CT) suggested a foreign body in the ileum with proximal small bowel dilatation. The object showed high-density outside and iso-density inside. The shape of the object was oval and 30 mm in diameter. The 3D construction image from the CT images showed a clearer shape of the object (Figure 2). 

**Figure 3:**
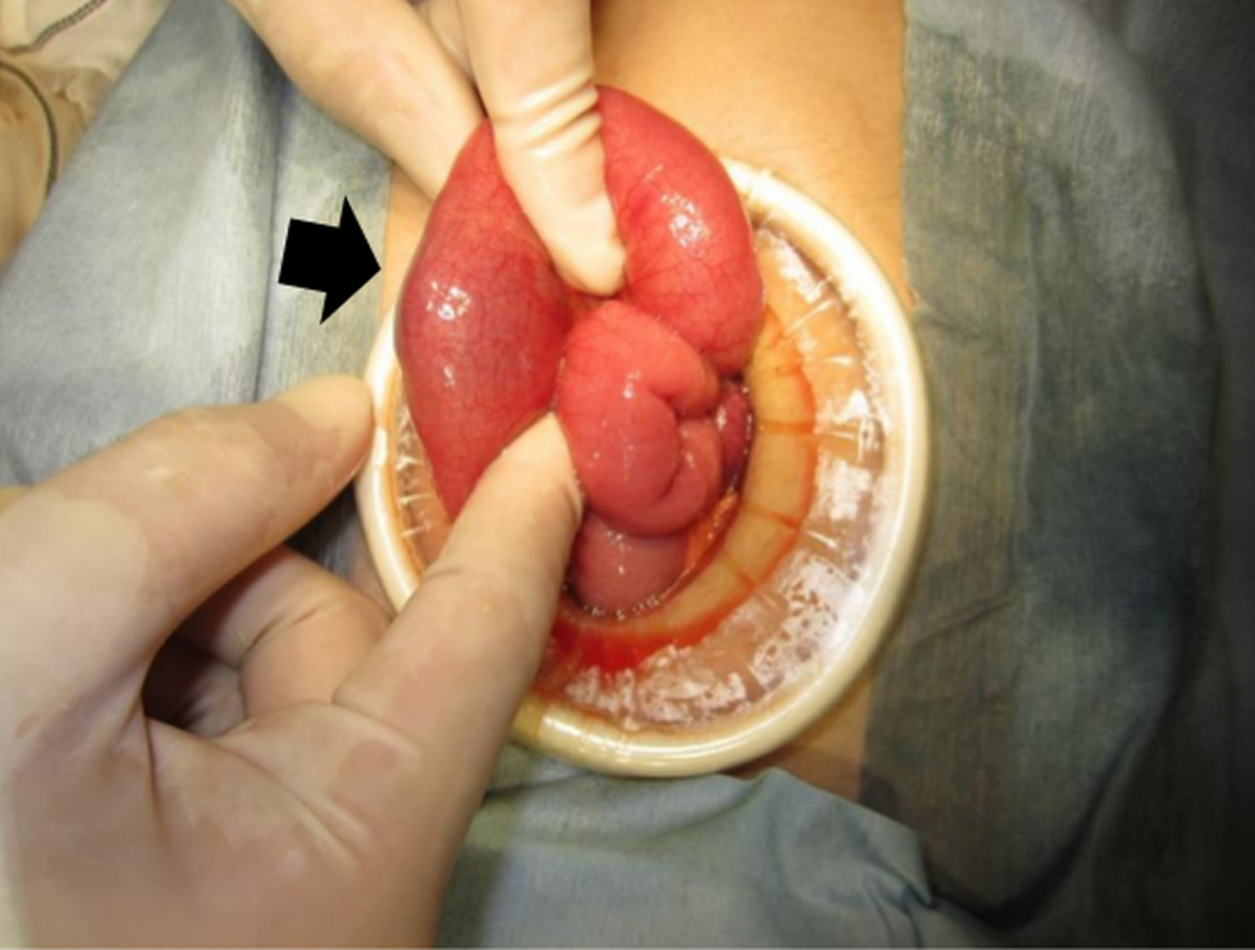
Operative findings The foreign body (arrow) was found impacted at the small intestine (approximately 20 cm from the oral side of the terminal ileum). Enterotomy was performed and the object was manipulated out of the small intestine.

Repeated interviews showed the following. The patient had eaten a whole peach eight days before visiting the hospital. Therefore, we diagnosed him with a small bowel obstruction caused by a seed. The foreign object was not expected to be discharged naturally because of its size. Therefore, we decided to perform surgical treatment. Intraoperatively, the foreign body was found impacted at the small intestine (approximately 20 cm from the oral side of the terminal ileum) (Figure 3).

**Figure 4:**
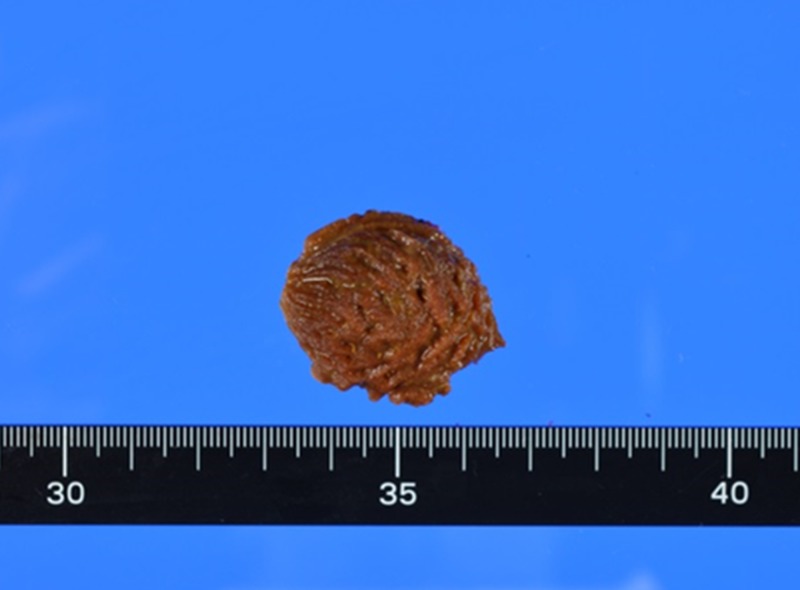
Foreign body Macroscopic examination of the foreign body. The foreign body was a peach seed and the size of the seed was 35×28 mm.

There was no major damage and no defects in the neighboring small intestine. Furthermore, there was no gastrointestinal disease in any other site of the small intestine. Enterotomy was performed proximally and the seed was manipulated out of the small intestine. The bowel condition was good (no damage or stenosis), and primary repair of the enterotomy site was performed. The diameter of the foreign body was 35×28 mm, and it was a peach seed, as expected (Figure 4).

The patient was discharged nine days after surgical treatment, with no perioperative complications.

## Discussion

Intestinal obstruction caused by plant seeds (IOPS) is rare because the shape of plant seeds is oval, and their size is small. There have been only four previous reports on IOPS [4-7]. Plant seeds sometimes form bezoars, but IOPS is a different disease from intestinal obstruction caused by bezoars in the clinical setting. Intestinal obstruction caused by bezoars has been reported [8]. However, bezoars can be formed from various objects (e.g., hair, fiber-rich vegetables) [9]. We found more reports on IOPS in Japan than in Europe and the United States. The reason for this finding could be due to differences in eating habits (e.g., Japanese people often eat Ume). We reviewed 29 reported cases of IOPS in Japan (Table 2; one English report and 28 Japanese reports).

**Table 2:** Review of reported cases of intestinal obstruction by plant seeds in the Japanese literature

Number of cases		29 (Male: 12, Female:17)		
Age		median 77.5 (range 15-91)		
Type of plant		Japanese apricot (Ume)	19	
		Loquat	4	
		Bayberry	1	
		Persimmon	3	
		Peach	2	
Site of obstruction		Ileum	18	
		Colon (right-side)	3	
		Colon (left-side)	7	
		Rectum	1	
Based gastrointestinal disease	Yes	Post-radiotherapy (RT)	9
		Colon cancer	10	
		Postoperative adhesion	1	
		Intestinal carcinoid	1	
		Crohn's disease	1	
		Anastomotic stenosis	2	
		Ischemic colitis	1	
		Intestinal Tb	2	
	No		2	

Importantly, 93.1% (27/29) of these patients had gastrointestinal disease in the site of the obstructed intestine. Only two cases without gastrointestinal disease were reported, including the current case [7]. In the healthy gastrointestinal tract, there can be four sites of obstruction: cardia, pylorus, ileocecal valve, and anus. The majority of foreign bodies that pass through the pylorus can be discharged to the outside from the anus naturally [2-3]. As described above, swallowed plant seeds are expected to be discharged naturally more often than other foreign bodies. The ileocecal valve of the young will be narrower than that of adults. This may be one of the reasons why the seed stagnated in the small intestine in our case.

Preoperative diagnosis of IOPS is not difficult because CT findings are characteristic of high density outside and low or iso-density inside the object. Therefore, when IOPS is diagnosed, we should consider that there may be gastrointestinal disease in the intestine.

## Conclusions

We report a rare case of IOPS without gastrointestinal disease. We wish to emphasize that IOPS without gastrointestinal disease is rare, but detailed interviews at a medical examination can identify this condition.
